# Harvard Odontological Society

**Published:** 1893-06

**Authors:** 

**Affiliations:** Harvard Odontological Society


### HARVARD ODONTOLOGICAL SOCIETY.

### DISCUSSION ON "HYPNOTISM."

(For the papers of Drs. Osgood and Fillebrown, see pages 337 and 355, respectively.)

*President Stanton.*—The Society is to be congratulated upon being so well enlightened upon the subject of hypnotism, or suggestive therapeutics. I am very curious about and always interested in new things. I have read Bernheim's work, and have had some experience in hypnotism, but there have been so many cases detailed this evening that I will not take up your time. I shall probably be called upon before a great while to read a paper, and I then may have something to say.

Dr. Osgood has kindly consented to answer any questions, and I will take advantage of the opportunity to ask him a few. First, in chronic cases of long standing, what are the first symptoms of success? In other words, what success does he generally have at the first sitting, and how many treatments, as a rule, does a chronic case require before it is comparatively cured?

Now, all of us, in presenting a subject, always show the favorable side; that is, a man presents his successes, never his failures. I think quite as much is to be learned from failures as from successes, and I should like to inquire about what proportion of the patients hypnotized are benefited by the hypnotic treatment?

Secondly, I should like to ask about what is the proportion of people who can be hypnotized; that is, can the majority, say eighty per cent., of people be hypnotized if time enough be taken?

I have noticed, in reading Bernheim's work, that the operators quoted nearly always give their successes at clinics or hospitals. Is it not possible that the nature of the education of that class of patients who go to hospitals may have something to do with their susceptibility? Or my third question may be put in this way, Will Dr. Osgood please tell us if the success in his private practice is as great as that obtained at a dispensary clinic?

*Dr. Osgood.*—Let me answer these numerous questions before I forget them. As I have already told you, a person's success with this treatment depends very much upon the patient himself. As Bernheim says, "Every man has his own way of being peculiar," and these peculiarities must be studied. A chronic case may show a great gain at once, and the first symptoms of success in the patient who gains gradually will be that after he has received treatment a few times he probably will say, "I feel that you are going to help me; I slept well last night, and feel a great deal better for it. I think my appetite is better, and I certainly feel sensations in that limb which are new to me," and from that time forward there is marked improvement.

The results of a first sitting vary greatly in different persons, and an acute case would probably show larger relief than would a chronic ailment. An affection of recent nature would be apt to disappear at once. It might require several sittings. A chronic malady might find striking relief from one application of suggestion, and afterwards recover more slowly. The number of treatments required differs with every case. There is no rule. As to the proportion of patients who receive benefit, I can say merely that relief is the rule. A small percentage of cases cannot be hypnotized; other patients are not adaptable. Cases, of course, present themselves which cannot be helped by any sort of treatment; but, where a patient is suggestible, the general result of the treatment is success.

I should think suggestion would be very useful in dentistry, because the operation is very slight; but where the operation is severe, the natural fear of it on the part of the patient might be so great as to predominate all other influence, and thus render suggestion of no avail. At the same time, all kinds of surgical operations can be and have been performed under the influence of hypnotism.

As to the number of times a chronic case would have to be treated, in comparison with the length of time given to a purely medical treatment, that would depend upon the susceptibility of the patient. I do not mean by this the degree of somnolence he reaches, for it is a curious fact that one patient will respond better in a light degree of sleep than others in a profound sleep. Patients differ so much in this respect that I cannot give a categorical answer to that question. One lady came to me who had suffered with headache for forty years and had tried everything that had been recommended by doctors and others as likely to help her. She was not suggestible, and I think I hypnotized her fifty times before she was sufficiently influenced to receive my suggestions to any extent, and she never had the same amount of headache afterwards. Another case that occurs to me was that of a gentleman who had always been in the habit of stammering, and came to me to see what I could do for him. I said to him, "Now, here is a beaten path which you have trod ever since you were a little boy, and it has become one of the regular habits of your life. It would be an effort for you to change your gait so that you could walk like a Prussian soldier, and so it will require days and days of treatment before I can make steady speech automatic with you. Your case is like that of a child who begins to learn an exercise on the piano, and who, at first, may say, 'Oh, I can never learn this,' but in the course of ten days he will play that piece and talk to some one at the same time." I hypnotized that gentleman about one hundred times, and the last time I saw him he said that he never stammered excepting when he was embarrassed. I will add that I advise those persons who have been cured of bad habits to come to me about once a month and be braced up.

Answering the question as to whether anybody can be hypnotized if time enough be given, there are records of extraordinary cases where patients have been hypnotized hundreds of times without being sufficiently influenced to receive suggestion. The most striking case of success seems to be one recorded by Beaunis, who tried every day for six months to put a lady to sleep, at the end of which period she one day fell into a profound slumber, and could always subsequently be hypnotized. But, you see, there is a question of money in this. I cannot give the time to a patient who is not easily affected, nor usually does the patient care to assume the expense. Ordinarily I try a difficult patient three or four times, and can then tell whether much time will be necessary in order to affect him. In such case the patient generally abandons the attempt. Out of one hundred patients, ten to fifteen cannot be influenced in the slightest degree. Bernheim himself failed to hypnotize a young lady who was a patient of mine, but whom I could influence to the first degree. She was abroad, and, simply out of regard for my interest in the treatment, took the trouble to go to Nancy. Bernheim did not influence her as much as I did. He is a man of extraordinary ability, but the lady had the idea when she went there that, as I had tried and Tuckey, of London, had tried, Bernheim probably would not do much better. This feeling may have prevented his success. Bernheim claims ability to affect, to one degree or another, ninety per cent. of his cases. I should say that in ordinary cases eighty per cent. ought to be influenced by every fair operator.

Concerning the relative proportion of success in private practice compared with that of the hospital, I am glad you mentioned that. It is smaller, because, all other things being equal, the cultivated mind is more difficult to reach than a mind which is simple and direct. A patient came to me last week from New York who was a very intelligent lady, and who had read somewhat on this subject; at the same time she believed that she could not be hypnotized, because yielding to hypnotic influence seemed to her a sign of mental weakness. I immediately suggested to her that as learned a man as Louis Agassiz became a perfect child under the influence of hypnotism, which evidently satisfied her mind on that point, because I had no trouble in putting her to sleep.

Another element which must be taken into consideration is the fact that in private practice one has a more independent class of people, and one must approach them in a different manner from that which one would use in a hospital, where the patients know they must obey orders or be sent away. Moreover, the hospital patient yields more readily than a private patient because he is influenced by seeing others about him fall asleep under suggestion.

*Dr. Briggs.*—Will Dr. Osgood please tell us if he does not sometimes meet an obstacle from the influence of outsiders who teach these suggestible people that there is danger to them in hypnotism, and if he does not find that such people are unnerved by it?

*Dr. Osgood.*—No; because while such a patient is asleep my suggestions that the treatment is harmless and helpful will impress the mind of the patient more than anything which may be said to him when he is awake. Even though that patient may be suggestible in the waking state, he would be still more so under the hypnotic influence. And if I found that some one was trying to make him believe that the treatment was dangerous, I should simply say while he was asleep, "You will not be affected by anything which is said to you on this subject while you are awake," and he would not be. But I have no such experiences.

*Dr. Clapp.*—I would like to know if Dr. Osgood has ever been under this suggestive influence himself?

*Dr. Osgood.*—No; I have not. I don't think I could be hypnotized. Liebeault, who has had enormous experience, says he has often been hypnotized by what he calls "fascination;" that is, the fixation of his own eyes put him to sleep instead of the patient. I have never been in any way affected by it.

*Dr. Smith.*—Is it possible to exert this influence on very young children?

*Dr. Osgood.*—It is admirable in children; the process is so simple, so direct, and so uncomplicated. Liebeault, who was a simple old man, says, "This really goes back to the days of the disciples. I find that by simply laying my hands on the children they become cured." This showed the power of suggestion, and if you will think of it you will see that suggestibility and suggestion rule us in all directions.

*Dr. Hopkins.*—I would like to ask Dr. Osgood if he has had success with this treatment on the slightly insane?

*Dr. Osgood.*—I have had several cases of fixed idea. One was a young lady who was very unhappy, because in church she imagined that the clergyman became embarrassed when he looked at her, and that he would become agitated if she spoke to him. She brooded over this a great deal, and it was finally decided that she should go away for a visit, and she came to Boston. While she was under hypnotic influence I told her that she must trust me; that the thing was now past and was nothing but a memory; that she should think nothing more about it; and I felt perfect confidence that when she got home she would find that I was right. After reaching home, she wrote me very gratefully, saying, "You are right; it is nothing but a memory, and does not disturb me any more." Another case was that of a lady who imagined that people made faces at her in the cars and on the street, and for that reason she disliked to go anywhere. I helped both those cases. Later, Dr. Cowles called me to the McLean Hospital to see a woman who had religious insanity. She would not touch a knife, fork, and spoon, nor three biscuits, three oranges, nor three articles of any kind, because they represented the Trinity to her, and when I was called she had refused to eat anything, because she said God did not wish her to eat, neither did He wish her to go out of doors. She was a fair subject and I succeeded quite well; but the next time I went to her, I unexpectedly met a very serious obstacle. She said that God had told her she was not to be hypnotized any more, and of course there was the end of it. In this direction, however, my experience has been limited. Voisin, of Paris, is the only man who has attained any degree of success with insane patients. But this is largely due to his enormous patience. He sometimes spends two hours upon a patient at one sitting. I cannot affect the insane as he does. Melancholia can be reached, but it is very hard to touch the fixed idea. That predominates everything.

*Dr. Smith.*—I would like to ask Dr. Osgood if it is not possible for him to influence patients that he has hypnotized when they are away from him?

*Dr. Osgood.*—Patients who are very susceptible, yes. But they must have been previously hypnotized, and the suggestion must reach them in a practical form. Patients have been put to sleep by the telephone and by mail. The sound of the voice of course makes the better suggestion, but if the doctor ends a note thus: "Now, when you finish this letter you will lay it down and sleep for half an hour," the patient will do so. Of course nobody can be influenced at a distance except by some one who has hypnotized him before, and the patient must be very suggestible.

*Dr. Shepherd.*—Are not many of the so-called "faith cures" something after the nature of hypnotism?

*Dr. Osgood.*—For information on that point I would refer you to a very interesting paper on "Psychical Research," in *Harper's Magazine* for June, 1890. In a foot-note the author virtually says, "This influence of mind upon mind explains the few successes of the faith cure, the Christian Science, and other methods of a similar nature practised by persons who have no medical knowledge, and treatment of this nature should be rescued from such hands and placed in the hands of people who have science to back them up." When Braid was teaching his patients he simply put them to sleep; but you see the patients came expecting to be helped, which was of itself a suggestion, and the result was they got well without a word. That is really the basis of the faith cure; it is a very weak form of suggestion, and through that suggestion comes a measure of relief. It may help the very susceptible cases which are merely hysterical, but you do not hear of its curing many cases of neuralgia, rheumatism, heart trouble, and so on. It is not hypnotic suggestion, and it does not include a necessary power of making an intelligent diagnosis.

Henry L. Upham, D.M.D.,
*Editor Harvard Odontological Society.*

